# Chemoenzymatic Synthesis of the New 3-((2,3-Diacetoxypropanoyl)oxy)propane-1,2-diyl Diacetate Using Immobilized Lipase B from *Candida* *antarctica* and Pyridinium Chlorochromate as an Oxidizing Agent

**DOI:** 10.3390/ijms21186501

**Published:** 2020-09-05

**Authors:** Esteban Plata, Mónica Ruiz, Jennifer Ruiz, Claudia Ortiz, John J. Castillo, Roberto Fernández-Lafuente

**Affiliations:** 1 Escuela de Química, Grupo de investigación en Bioquímica y Microbiología (GIBIM), Edificio Camilo Torres 210, Universidad Industrial de Santander, CEP, 680001 Bucaramanga, Colombia; stbnplata29@gmail.com (E.P.); icmonicaruiz@gmail.com (M.R.); jennifer.ruiz@correo.uis.edu.co (J.R.); 2 Escuela de Microbiología, Universidad Industrial de Santander, 680001 Bucaramanga, Colombia; ortizc@uis.edu.co; 3 ICP-CSIC, Campus UAM-CSIC, Cantoblanco, 28049 Madrid, Spain

**Keywords:** chemoenzymatic synthesis, glycerol derivatives, interfacially activated lipase, regioselective hydrolysis, diacetin oxidation, pyridinium chlorochromate, antibacterial activity, antifungal activity, hemolytic activity

## Abstract

To exploit the hydrolytic activity and high selectivity of immobilized lipase B from *Candida antarctica* on octyl agarose (CALB-OC) in the hydrolysis of triacetin and also to produce new value-added compounds from glycerol, this work describes a chemoenzymatic methodology for the synthesis of the new dimeric glycerol ester 3-((2,3-diacetoxypropanoyl)oxy)propane-1,2-diyl diacetate. According to this approach, triacetin was regioselectively hydrolyzed to 1,2-diacetin with CALB-OC. The diglyceride product was subsequently oxidized with pyridinium chlorochromate (PCC) and a dimeric ester was isolated as the only product. It was found that the medium acidity during the PCC treatment and a high 1,2-diacetin concentration favored the formation of the ester. The synthesized compounds were characterized using IR, MS, HR-MS, and NMR techniques. The obtained dimeric ester was evaluated at 100 ppm against seven bacterial strains and two *Candida* species to identify its antimicrobial activity. The compound has no inhibitory activity against the bacterial strains used but decreased *C. albicans* and *C. parapsilosis* growth by 49% and 68%, respectively. Hemolytic activity was evaluated, and the results obtained support the use of the dimeric ester to control *C. albicans* and *C. parapsilosis* growth in non-intravenous applications because the compound shows hemolytic activity.

## 1. Introduction

Glycerol (propane-1,2,3-triol) is the most abundant by-product generated during transesterification reactions of vegetable oils and animal fats with methanol to produce biodiesel [1,2,3]. Due to the global population growth and the demand for new energy sources with a low impact regarding CO_2_ emissions [4,5], the biodiesel industry is developing rapidly, resulting in a high excess of glycerol. Therefore, new processes to convert this alcohol into added-value chemicals have been increasingly explored [6,7,8,9].

A well-known process for the transformation of glycerol is its esterification or transesterification to monoglycerides, diglycerides, and triglycerides [10,11], molecules that exhibit high nutritional value [12], and are extensively used as emulsifiers in the food, cosmetic, and pharmaceutical industries [13]. At an industrial scale, glycerides are generally prepared using inorganic acid or basic catalysts at high temperatures [14] or by enzyme-catalyzed [15] reactions, such as alcoholysis, glycerolysis, and hydrolysis [16], which, unlike chemical methods, can afford glycerides with high yields and selectivity under mild conditions in the absence of by-products [17,18].

Enzymes are very useful catalysts for organic chemistry because they can be used in a wide range of reactions, and also because they show high reaction rates and selectivity [19]. Amongst the enzymes, lipases have been found to be particularly useful [20,21]. In nature, lipases have the physiological function of hydrolyzing fats and oils. However, in vitro, they can be used in other processes such as amidation [22,23] and esterification [24] reactions. Their high enantiospecificity also makes them suitable for enantiomeric resolution processes [25,26]. Due to their regioselectivity and the fact that they can be used under mild conditions in aqueous and organic media [27,28], lipases are preferred as biocatalysts to modify glycerol and its derivates selectively.

Glycerides are molecules that have low water solubility; therefore, they form insoluble drops where lipases act. This action of lipases at the interfaces is the peculiarity of these enzymes. This capacity of lipases is possible due to their mechanism of action, called interfacial activation, which permits lipases to become adsorbed on the hydrophobic surface of the glyceride drops by involving their active center area and, that way, they can act at the interface of these oil drops. In homogeneous media, the active center of lipases is generally isolated from the reaction medium because it is covered with a polypeptide chain called lid (in this “closed” form, the lipase is usually inactive). This lid can move, and when it is shifted, it forms a huge hydrophobic pocket, exposing the active center to the medium, resulting in the “open” and active form of the lipase, with the hydrophilic phase of the lid interacting with areas of the protein surface near the active center [29,30,31]. This large hydrophobic pocket is very unstable in homogeneous aqueous media. Both conformational lipase forms are in equilibrium, but in the presence of oil drops, the open form becomes adsorbed on the hydrophobic surface of the drops, shifting the conformational equilibrium towards the open form of the lipase and permitting the attack of the glycerides by the enzyme [29,32,33]. This phenomenon has been exploited to immobilize lipases on hydrophobic supports, such as octyl-agarose, which has octyl groups on its surface and can selectively immobilize lipases via interfacial activation. Lipase immobilization is essential in all industrial applications because it allows the separation and reuse of the biocatalyst; studies have shown that immobilization on supports via interfacial activation lends stability, improves selectivity, and enhances catalytic activity [32,34,35,36,37].

Oxidation is another way to prepare added-value compounds from glycerol, fine chemicals such as dihydroxyacetone, glyceric acid, and glyceraldehyde, which have found applications in the cosmetics and pharmaceutical industries [38,39,40,41]. Considering the three functional groups present in the glycerol molecule (two primary and one secondary hydroxyl groups), selective oxidation of these functionalities is the main aspect to consider when selecting methodologies to prepare the aforementioned molecules, which are usually prepared using expensive metals such as palladium, gold, and platinum as catalysts [42,43,44]. Chromium (VI)-based oxidants, such as the Collins reagent [45], pyridinium chlorochromate [46], or pyridinium dichromate [47], have been extensively used as oxidizing agents in organic synthesis, due to their efficiency and most importantly because oxidation of alcohols using such types of reagents allows the user to stop the reaction at the aldehyde or carboxylic acid stage if the reaction is properly controlled [48,49].

Glycerol can undergo oligomerization by etherification reactions: two glycerol molecules can condensate to yield diglycerols, which may be linear, branched, or cyclic [50,51]. Low molecular weight glycerol oligomers (PGs) and polyglycerol esters (PGEs) have found applications in the cosmetic, pharmaceutical, and food industries as new products for surfactants, lubricants, cosmetics, and food additives [52,53,54,55,56]. PGs and PGEs are normally obtained from the high temperature etherification of glycerol and the esterification with fatty acids in the presence of homogenous or heterogenous catalyst [57,58,59,60].

To exploit the regioselectivity of the immobilized lipases and the efficiency of the chromium (VI) oxidants, in this work we propose a new chemoenzymatic methodology in which we used *Candida antarctica* lipase B immobilized on octyl-agarose support (CALB) to hydrolyze triacetin to 1,2-diacetin, which was subsequently transformed, using PCC, to obtain the new dimeric glycerol ester 3-((2,3-diacetoxypropanoyl)oxy)propane-1,2-diyl diacetate, a molecule which to date has no reported synthesis. To the best of our knowledge, this is the first time that a diglyceride prepared via catalytic hydrolysis with immobilized lipases has been used as the starting material for new and more complex molecules.

## 2. Results and Discussion

### 2.1. Immobilization of CALB on Octyl-agarose

**Figure 1:**
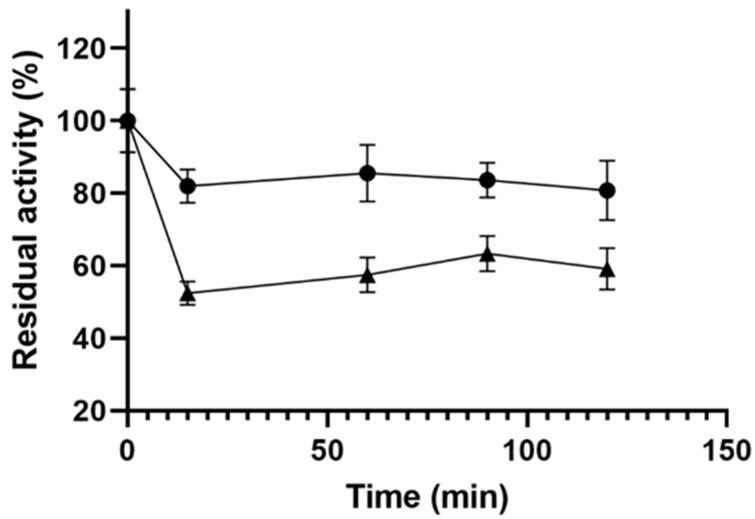
Immobilization course of *Candida antarctica* lipase B (CALB) on octyl agarose. (●) suspension and (▲) supernatant.

Figure 1 shows the immobilization course of CALB on octyl-agarose, which is a quite rapid immobilization process. As we used an excess of the enzyme, immobilization only accounts for around 50% of the offered enzyme, with an expressed activity of around 50% [61].

Compared to other lipases [62,63], in our case there was no hyperactivation of the enzyme upon immobilization, probably since CALB has no large helical lid fully covering the active site and does not exhibit a strong interfacial activation [33,64,65]; therefore, its activity in solution is very similar to the activity of the enzyme immobilized on octyl-agarose [66,67,68], and even decreased due to substrate diffusional problems by the high enzyme loading.

### 2.2. Hydrolysis of Triacetin (1) Catalyzed by CALB-OC

**Figure 2:**
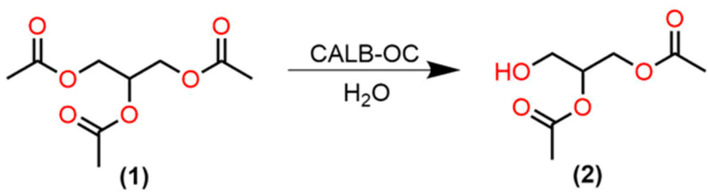
Regioselective hydrolysis of triacetin (1) to 1,2-diacetin (2) catalyzed by CALB-OC.

Figure 2 shows the enzymatic hydrolysis of triacetin catalyzed by CALB-OC. In this study, we use CALB-OC, since it has been shown that, like most lipases, CALB-OC is a *sn*-1,3 regioselective biocatalyst in the hydrolysis of triacetin, so that the hydrolysis of the triglycerides with lipases will produce 1,2-diglycerides and 2-monoglycerides [69,70,71]. It is interesting to note that, based on previous studies, hydrolysis of triacetin by Novozym 435 (a commercial immobilized preparation of CAL B) exhibited a low yield of diacetin [72], presumably due to high isomerization and production of monoacetins [73,74].

To assess the effect of acetonitrile on the hydrolysis of triacetin by CALB-OC and to improve the solubility of triacetin, we evaluated the addition of 20% acetonitrile to the reaction mixture. Table 1 shows the yield of 1,2-diacetin (2) in the absence and the presence of 20% acetonitrile (entry 1). After 90 min of reaction, 1,2-diacetin (2) and 2-monoacetin were the only detected products and 46.5% of the triacetin remained non-hydrolyzed. On the other hand, the hydrolysis without acetonitrile (entry 2) increased the yield of 1,2-diacetin (2) to 71% with an increase in the accumulation of 2-monoacetin. 1,3-diacetin was not detected in any of the two cases [75]. The reduction in the production of 1,2-diacetin (2) in the presence of acetonitrile can be attributed to the fact that organic solvents alter the conformation of the immobilized lipases [76] and could also act as competitive lipase inhibitors [69].

We isolated 1,2-diacetin (2) from 2-monacetin by a liquid–liquid separation in which 2-monacetin remained in the aqueous phase and 1,2-diacetin (2) in the organic phase. To ensure complete hydrolysis of triacetin (1), we increased the reaction time to 3 h; finally, a purified 1,2-diacetin (2) structure was corroborated by infrared spectroscopy, ^1^H NMR and, GCMS (all spectra are found in the Supporting Information, Figures S1–S3).

### 2.3. 1,2-Diacetin (2) Oxidation with PCC

**Figure 3:**
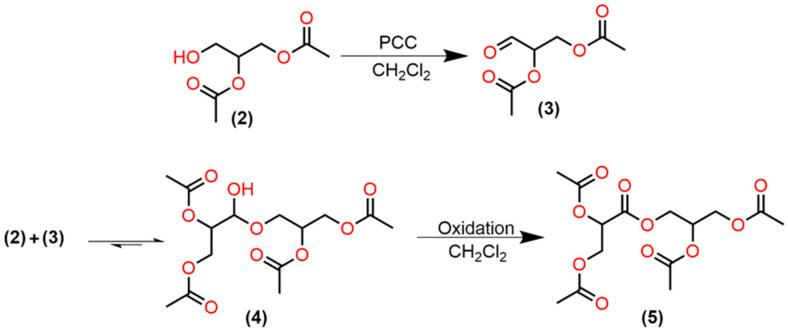
1,2-diacetin (2) oxidation and dimeric ester (5) formation; (3) glyceraldehyde diacetate, (4) dimeric hemiacetal.

To propose new methodologies for the synthesis of glycerol-derived compounds, we attempted to oxidize 1,2-diacetin (2) to glyceraldehyde diacetate (3) (Figure 3) using 2 equivalents of PCC in dichloromethane at 25 °C for 12 h (Table 2, entry 1). GCMS analysis showed the formation of the expected glyceraldehyde diacetate (3) (mass spectrum is shown in the Supporting Information, Figure S4) and another compound of higher molecular weight. After filtration through a silica pad, glyceraldehyde diacetate (3) vanished from the reaction crude. Further analysis of the remaining product showed that its structure corresponds to 3-((2,3-diacetoxypropanoyl)oxy)propane-1,2-diyl diacetate (5) (all spectra can be found in the Supporting Information, Figures S5–S9).

The formation of ester (5) can be explained if we take into account that the acidity of PCC can promote a rapid addition of 1,2-diacetin (2) to the glyceraldehyde diacetate (3) to form the dimeric hemiacetal (4), from which subsequent oxidation produces the dimeric ester (5) (Figure 3) [77,78,79].

To increase the yield of the glyceraldehyde diacetate (3) and to avoid the formation of the hemiacetal (4), which could be promoted by the acidity of PCC, we carried out the reaction adding two equivalents of sodium acetate [80] (Table 2, entry 2) and it was found that decreasing the acidity of the medium promotes the accumulation of the aldehyde (3) with a consequent decrease in the reaction rate. Slow addition of 1,2-diacetin (2), and a higher solvent/PCC ratio (Table 2, entry 3), avoids the formation of ester (5); probably the low concentration of alcohol (2) slows the formation of hemiacetal (4) [78]. Accordingly, the best aldehyde proportions were obtained when the reaction was carried out adding only 1 equivalent of sodium acetate and simultaneously reducing the 1,2-diacetin (2) concentration by slow addition and high dilution (Table 2, entry 4) (all chromatograms and mass spectra are shown in the Supporting Information, Figures S10–S16).

**Figure 4:**
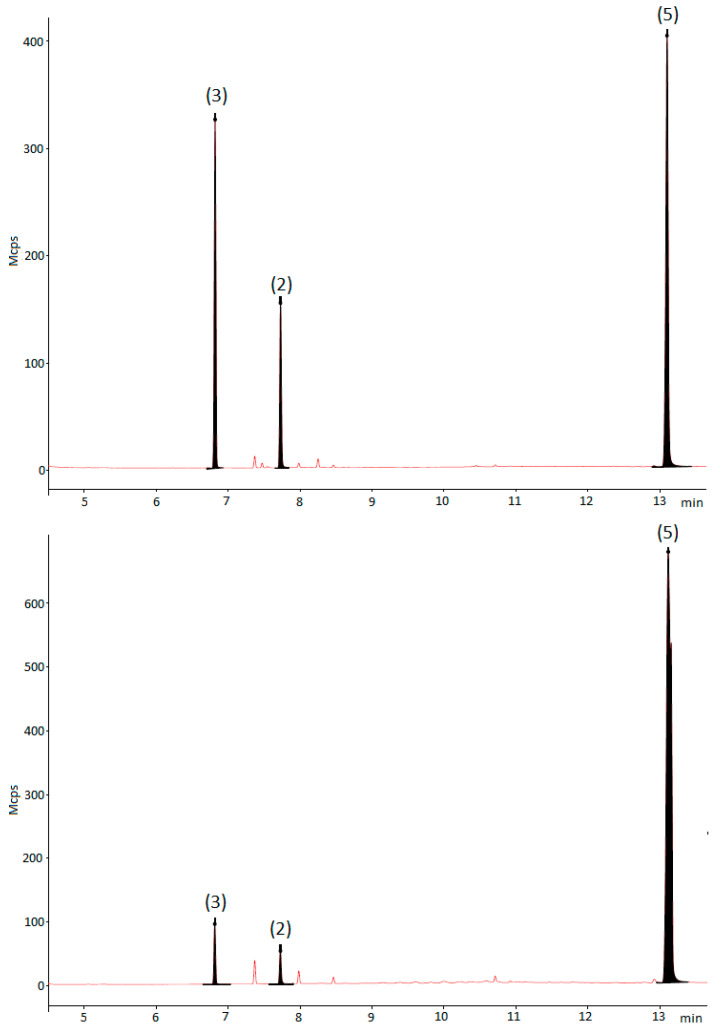
Effect of the reaction workup over the crude composition. Compound notation: (3) glyceraldehyde diacetate, (2) 1,2-diacetin, and (5) 3-((2,3-diacetoxypropanoyl)oxy)propane-1,2-diyl diacetate. Top: GC analysis of the reaction crude performed before the reaction workup. Bottom: GC analysis performed after the reaction’s crude filtration through a silica pad.

It is worth mentioning that glyceraldehyde diacetate (3) could never be isolated for the NMR analysis because the reaction workup promotes the accumulation of the ester (5). Figure 4 shows the reaction’s crude (using conditions shown in Table 2 entry 4) composition before and after filtration through a silica pad. During the reaction workup, the unreacted 1,2-diacetin and the produced glyceraldehyde diacetate reacted, to generate compound (5) as the major product in the reaction, no matter which reaction conditions are initially used. We evaluated the effect of silica gel and found that it promotes the accumulation of the dimeric ester (5) (Table 2 entry 5). If celite [82] is used for filtration, the reduced chromium species are not eliminated from the reaction crude; consequently, a silica gel pad must always be used for filtration [83,84].

### 2.4. Biological Activity of 3-((2,3-Diacetoxypropanoyl)oxy)propane-1,2-diyl diacetate (5)

Antimicrobial activity against MRSA, *S. aureus* ATCC 29213, *E. coli* ATCC 25922, *E. coli* O157:H7, *S. typhimurium* ATCC 14028, *S. enteritidis* ATCC 13076, *P. aeruginosa* ATCC 27853, *C. albicans* ATCC 10231, and *C. parapsilosis* ATCC 22019 was evaluated. 3-((2,3-diacetoxypropanoyl)oxy)propane-1,2-diyl diacetate does not show growth inhibitory activity at 100 ppm in any of the bacterial strains. However, an increase in the growth of *E. coli* O157: H7 and *Salmonella* spp. was evidenced. On the other hand, evaluation of the fungal activity of the dimeric ester showed growth inhibition of both *Candida* strains at 100 ppm; the strain *C. parapsilosis* being the most sensitive with a 68% inhibition within 48 h of culturing. Finally, hemolytic activity was evaluated; the new compound presented hemolysis of 18.5 ± 2.6% at a concentration of 100 ppm, which is considered high, taking into account that a percentage of hemolysis greater than 10% is considered hemolytic or not hemocompatible [85] (biological activity can be found in the Supporting Information, Figures S17–S19).

To date, this is the first report that describes the synthesis of a new glycerol derivative obtained by enzymatic production of 1,2-diacetin and its posterior oxidation with PCC. The dimeric glycerol ester (5), whose synthesis is herein reported, could be of use for the cosmetics, pharmaceutical, and food industries, and could serve as the starting material for the synthesis of new molecules.

## 3. Materials and Methods

Soluble CALB was kindly donated by Novozymes. The octyl Sepharose 4BCL beads were obtained from Sigma Aldrich. Pyridinium chlorochromate was prepared using the Corey and Suggs protocol [46]. Silica gel, 230–400 mesh, molecular sieves of 0.4 nm, dichloromethane, n-hexane, ethyl acetate, and diethyl ether were obtained from Merck. The *p*-nitrophenylbutyrate (*p*-NPB) was obtained from Sigma Aldrich. Reaction monitoring was performed using silica gel TLC plates (silica Merck 60 F254), and the spots were visualized using Vanillin-HCl staining. Reaction monitoring was also performed using HPLC-DAD (Agilent Santa Clara, CA, United States) with a Zorbax C-18 (5 µm × 250 mm × 4.6 mm) column. ^1^H and ^13^C NMR spectra were measured at 25 °C on a Bruker Advance III–400 spectrometer, using CDCl_3_ as the solvent. Chemical shifts (δ) and coupling constants (J) values are reported in ppm and Hz, respectively. Chemical shifts are relative to the solvent peaks used as reference (CDCl_3_: δ 7.26 for 1H and δ 77.23 for ^13^C). ^1^H NMR assignments were d = doublet, s = singlet, br = broad, and m = multiplet. Gas chromatograms and low-resolution mass spectra were recorded using a ZB-5 ms 30 m × 0.25 mm × 1.0 µm and a 15 m × 0.25 mm × 1.0 µm column on a Bruker EVOQ GC-TQ (Bruker Billerica, MA, United States) gas chromatograph (EI: 70 eV, full scan); temperature program: 1 min at 40 °C, 40–150 °C heating at 30 °C/min, 1 min at 150 °C, 150–250 °C heating at 15 °C/min, and 3 min at 250 °C. High-resolution mass spectra were recorded on a Waters Micromass AutoSpect NT operating at 70 eV. UHPL-HRMS data were obtained on a Dionex Ultimate 3000 chromatograph accoupled to an Orbitrap Exactive Plus spectrometer via electrospray ionization. Strains of MRSA and *E. coli* O157:H7 were acquired from a microorganism collection by the Pontificia Universidad Javeriana from Colombia (CMPUJ-certified by the World Federation of Culture Collection). *Candida* strains were donated by the School of Microbiology of Universidad Industrial de Santander. *S. aureus* ATCC 29213, *E. Coli* ATCC25922, *P. aeruginosa* ATCC 27853, *S. enteriditis* ATCC13076, and *S. Typhimurium* ATCC14028 were obtained from ATCC.

### 3.1. Standard Activity Determination

*p*-NPB hydrolysis was used as a model reaction to determine the standard activity during immobilization. A 20–100 µL lipase suspension or solution was added to 2.5 mL of 25 mM sodium phosphate at pH 7 and 25 °C and the increase in absorbance at 348 nm produced by the release of *p*-nitrophenol during the hydrolysis of 0.4 mM *p*-NPB was measured [86]. One international unit of activity (U) was defined as the amount of enzyme that hydrolyzes 1 µmol of *p*-NPB per minute under the conditions described previously. The protein concentration was determined by Bradford’s method using BSA as standard [87].

### 3.2. Immobilization of CALB on Octyl agarose beads

CALB was immobilized on octyl agarose beads at low ionic strength as previously described [34]. A total of 1 g of octyl sepharose support resuspended in 20 mL of an enzymatic solution (containing 0.54 mg/mL of protein and specific activity 10.57 U/mg) was gently stirred in a shaker at 250 rpm in phosphate buffer sodium (5 mM and pH 7). The activities of both the supernatant and suspension were followed using a *p*-NPB assay. After the indicated time, the immobilized enzyme (protein concentration 4 mg/g of CALB-OC, specific activity 15.5 U/mg) was recovered by being filtered and washed several times with distilled water (3 × 20 mL).

### 3.3. Triacetin (1) Hydrolysis with CALB-OC

A total of 3.8 g of CALB-OC were stirred for 3 h with a solution of 1.9 mL (9.95 mmol) triacetin in 100 mL of 500 mM sodium phosphate at pH 5.5 at room temperature. Upon completion of the reaction monitored by HPLC, the biocatalyst was filtered, washed with 30 mL of distilled water, and stored for reuse. The filtrate was saturated with NaCl and extracted with dichloromethane (3 × 30 mL), the organic layer was washed with brine (1 × 20 mL) and dried over sodium sulfate, the solvent was removed under reduced pressure to afford 1.297 g (yield: 74%, 7.36 mmol) of 1,2-diacetin as a colorless liquid. IR (ATR): 3462 (O-H), 2957 (C-H), 1734 (C=O), 1217 (C-O ester), 1043 (C-O alcohol). ^1^H NMR: δ = 5.09 to 5.03 (m, 1H, 2-CH), 4.34-4.17 (m, 2H, 3-CH_2_), 3.72 (d, J = 5.1 Hz, 2H, 1-CH_2_), 2.39 (br s, 1H, 1-OH), 2.09 (s, 3H, 3-OAc), 2.06(s, 3H, 2-OAc). MS (EI, 70 eV): *m/z* (%) 145 (11), 103 (15), 43 (100).

### 3.4. Synthesis of 3-((2,3-Diacetoxypropanoyl)oxy)propane-1,2-diyl diacetate (5)

A total of 2.44 g (11.35 mmol) of PCC, 1 g of activated molecular sieves, and 23 mL of dichloromethane were stirred for 10 min in a round bottom flask, and then a solution of 1 g (5.68 mmol) of 1,2-diacetin in 5 mL of dichloromethane was added and the mixture was stirred at 25 °C for 12 h. Upon completion of the reaction monitored by TLC, 10 mL of diethyl ether were added to the reaction mixture, which was later filtered through a pad of silica and washed with diethyl ether (3 x10 mL). The solvents were removed under reduced pressure and the remaining crude was purified by column chromatography in silica gel using a 5:2 hexane:ethyl acetate as eluent to afford 0.623 g (yield 63%, 1.79 mmol) of the product as a colorless liquid. ^1^H NMR: δ = 5.32 to 5.24 (m, 2H, 2-CH, 2′-CH), 4.5 to 4.3 (m, 4H, 2′-CH_2_, 3′-CH_2_), 4.3 to 4.1 (m, 2H, 3-CH_2_), 2.17 (s, 3H, 3-OAc), 2.09 (s, 6H, 1′-OAc, 2′-OAc), 2.08 (s, 3H, 2-OAc). ^13^C NMR: δ = 170.58, 170.48, 170.21, 170.13, 167. 08, 70.38, 68.81, 63.63, 62.60, 62.15, 20.96, 20.81, 20.78, 20.63. MS (IE, 70 eV): *m/z* (%) 173 (17), 159 (12), 145 (13), 131 (7), 103 (15), 43 (100). HRMS (ESI+): *m/z* [M+H]^+^ Calculated for C_14_H_21_O_10_: 349.11347, found: 349.11307, *m/z* [M+NH_4_]^+^ Calculated for C_14_H_24_O_10_N 366.1400, found, 366.13953.

### 3.5. Biological Activity of 3-((2,3-Diacetoxypropanoyl)oxy)propane-1,2-diyl diacetate (5)

#### 3.5.1. Antibacterial Activity

The antimicrobial activity of 3-((2,3-diacetoxypropanoyl)oxy)propane-1,2-diyl diacetate (5) was evaluated by the micro dilution method described in previous works [88,89]. Briefly, a pre-inoculum in Luria Bertani (*E. coli* ATCC 25922, *E. coli* O157:H7, *S. typhimurium* ATCC 14028, *S. enteritidis* ATCC 13,076, and *P. aeruginosa* ATCC 27853) and in Mueller Hinton (MH) for MRSA and *S. aureus* ATCC 29213, were grown at 37 °C during 12 h at 200 rpm; then, the culture of each strain was set at 0.5 in the McFarland scale (10^5^ CFU mL^−1^); 100 μL aliquots of these cell suspensions were mixed with 100 μL of compound (5) at 100 ppm in a 96-well microplate and incubated at 37 °C in an orbital shaker (200 rpm for 8 h). The bacterial growth kinetics of these microbial cultures was performed measuring changes of absorbance at 595 nm over time in an Elisa reader for these cultures (Thermo Fisher Scientific Waltham, MA, United States). MIC_50_ was defined as the lowest concentration of dimeric ester (5), inhibiting 50% of the bacterial growth of these bacterial strains. After incubation for 8 h, 100 μL of these bacterial cultures were poured in 900 μL of BHI, incubated at 37 °C for 24 h, and then a 10 μL of these cultures were seeded over BHI-agar petri dishes, incubated for 1 day at 37 °C, after which the appearance of colonies were determined. The MBC was the lowest concentration producing a >99.9% reduction in colony-forming units (CFU).

#### 3.5.2. Antifungal Activity

The antifungal activity of 3-((2,3-diacetoxypropanoyl)oxy)propane-1,2-diyl (5) diacetate was evaluated over *C. parapsilosis* ATCC 22,019 and *C. albicans* ATCC 10,231 by the microdilution method described in previous works [90]. From a fresh culture, a suspension was adjusted to an absorbance of 0.09–0.13 at 490 nm in sterile saline solution (10^6^ CFU mL^−1^). Once adjusted, a dilution was carried on Roswell Park Memorial Institute medium (RPMI) to obtain a concentration of 10^3^ CFU mL^−1^, which was inoculated in 96-well plates with 100 ppm of compound (5) and incubated at 37 °C for 48 h. Once the incubation time was over, the absorbance at 490 nm was measured in a microplate reader (Thermo Scientific™, Multiskan Sky) and compared with the selected growth controls.

#### 3.5.3. Hemolytic Activity

The hemolytic activity of compound 3-((2,3-diacetoxypropanoyl)oxy)propane-1,2-diyl diacetate (5) on sheep erythrocytes was evaluated, adapting the method described in previous works [91,92]. From 1 mL of defibrinated blood, three serial washes were carried out with 0.9% saline solution and the cell density was adjusted to 1% *v/v* by using cell counting in a Neubauer chamber. Then, 100 µL aliquots of the adjusted suspension were transferred to 96-well microplates and incubated with one containing a 100 ppm solution of compound (5), and were incubated for 3 h at 37 °C. The microplates were centrifuged at 500× *g* in 5 min and the absorbance of the released hemoglobin was measured at 543 nm using a microplate reader (Thermo Scientific™, Multiskan Sky). Saline solution and 0.5% triton X-100 were used as the negative and positive controls, respectively. To determine the percentage of hemolysis, Equation (1) was used.

(1) $${\%}_{\mathrm{hemolysis}}=\frac{\left(\mathrm{Am}-\mathrm{Acn}\right)}{\left(A_{\mathrm{Cp}}-A_{\mathrm{cn}}\right)}\;\times \;100$$

where Am is the absorbance of the sample, Acp is the absorbance of the positive control, and Acn is the absorbance of the negative control.

## 4. Conclusions

In this work, we have shown that immobilized lipases are very useful tools for the synthesis of new glycerol value-added derivatives. We developed a chemoenzymatic methodology for the synthesis of a new glycerol dimeric ester, in which we made use of CALB immobilized on octyl-agarose, which proved to be an efficient biocatalyst for the regioselective hydrolysis of triglycerides, to synthesize under mild conditions 1,2-diacetin, which was subsequently oxidized with PCC. Interestingly, this oxidation did not afford the expected aldehyde but a dimeric ester through a mechanism that involves the condensation of the starting diglyceride and the product aldehyde. Finally, the dimeric glycerol ester obtained has antifungal activity; however, it does not show activity on bacterial growth, which allows us to establish that it is a biologically active compound on eukaryotic cells. The hemolytic activity was evaluated, and the results obtained support the use of the dimeric ester to control *C. albicans* and *C. parapsilosis* growth in non-intravenous applications because the compound produces hemolysis at the evaluated concentration. 

## Figures and Tables

**Table 1:** Effect of solvents on the triacetin hydrolysis with CALB-OC.

Entry	Solvent	Triacetin (%)	1,2-Diacetin (%)	2-Monoacetin (%)
1	20% acetonitrile/sodium phosphate 500 mM pH 5.5	46.5	43.6	9.9
2	Sodium phosphate 500 mM pH 5.5	5.4	71.0	23.6

Percentage composition of glycerides after 90 min of reaction.

**Table 2:** Effects of the reagent and solvent/PCC ratio on 1,2-diacetin oxidation *.

Entry	Reagent	Solvent/PCC Ratio	Ratio (GC) (2):(5):(3) **
1	PCC 2 equiv	10 mL CH_2_Cl_2_/g PCC	4:6:1
2	PCC 2 equiv, AcONa 2 equiv	10 mL CH_2_Cl_2_/g PCC	14:1:3
3	PCC 2 equiv	20 mL CH_2_Cl_2_/g PCC	3:8:4
4	PCC 2 equiv, AcONa 1 equiv	20 mL CH_2_Cl_2_/g PCC	1:4:2
5	PCC 2 equiv, AcONa 1 equiv, silica gel 2 g	20 mL CH_2_Cl_2_/g PCC	1:5:1

* 3 Å molecular sieves [81] were used in all reactions at room temperature for 12 h. ** GC analyses were performed before the reaction workup, as described in Section 3.

## Supplementary Materials

Supplementary Materials Figures S1–S19 are available online at https://www.mdpi.com/1422-0067/21/18/6501/s1.

## Abbreviations

CALB	*Candida antarctica*
CALB-OC	*Candida antarctica* immobilized on octyl-agarose
PCC	pyridinium chlorochromate
IR	infrared spectroscopy
NMR	nuclear magnetic resonance
MS	mass spectrometry
HR	high resolution
PGs	polyglycerols
PGEs	polyglycerol esters
MIC_50_	minimal inhibitory concentration
MRSA	methicillin-resistant *Staphylococcus aureus*
