# Supplementary material for: Chemoenzymatic Synthesis of the New 3-((2,3-Diacetoxypropanoyl)oxy)propane-1,2-diyl Diacetate Using Immobilized Lipase B from Candida antarctica and Pyridinium Chlorochromate as an Oxidizing Agent
Source: Int J Mol Sci. 2020 Sep 5;21(18):6501. doi: 10.3390/ijms21186501 (PMC7555366; doi:10.3390/ijms21186501)

Supporting Information

Chemoenzymatic Synthesis of the New 3-((2,3-diacetoxypropanoyl)oxy)propane-1,2-diyl Diacetate Using Immobilized Lipase B from *Candida* *antarctica* and Pyridinium Chlorochromate as an Oxidizing Agent

Esteban Plata ^1^, Mónica Ruiz ^1^, Jennifer Ruiz ^1^, Claudia Ortiz ^2^, John J. Castillo ^1,^* and Roberto Fernández-Lafuente ^3,^*

^1^ Escuela de Química, Grupo de investigación en Bioquímica y Microbiología (GIBIM), Edificio Camilo Torres 210, Universidad Industrial de Santander, CEP,680001 Bucaramanga, Colombia; stbnplata29@gmail.com (E.P.); icmonicaruiz@gmail.com (M.R.); jennifer.ruiz@correo.uis.edu.co (J.R.)

^2^ Escuela de Microbiología, Universidad Industrial de Santander, Bucaramanga, Colombia; ortizc@uis.edu.co

^3^ ICP-CSIC. Campus UAM-CSIC, Cantoblanco, 28049 Madrid, Spain

***** Correspondence: jcasleon@uis.edu.co (J.J.C.); rfl@icp.csic.es (R.F.-L.); Tel.:+57-320-902-6464 (J.J.C.); +34915854804 (R.F.-L.)

Received: 3 August 2020; Accepted: 4 September 2020; Published: date

Figure S1. IR SPECTRUM OF 1,2-DIACETIN (2).


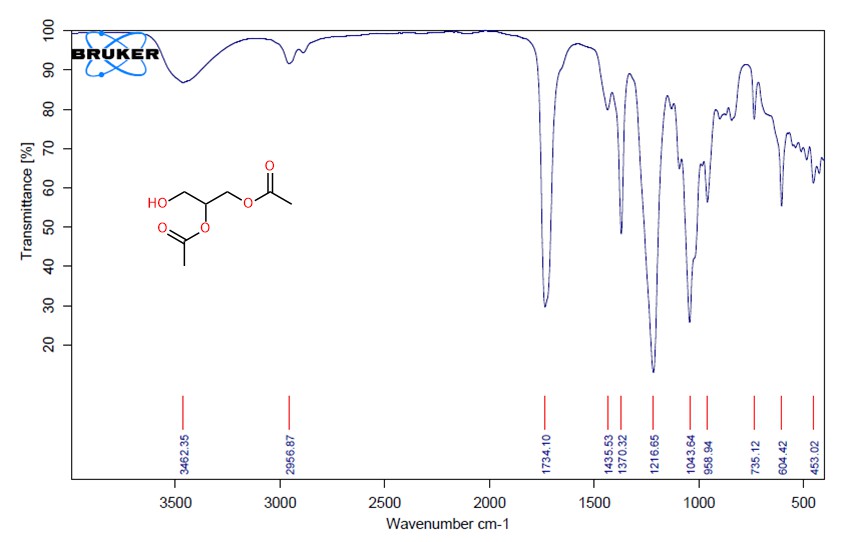


Figure S2. ^1^H NMR SPECTRUM OF 1,2-DIACETIN (2).


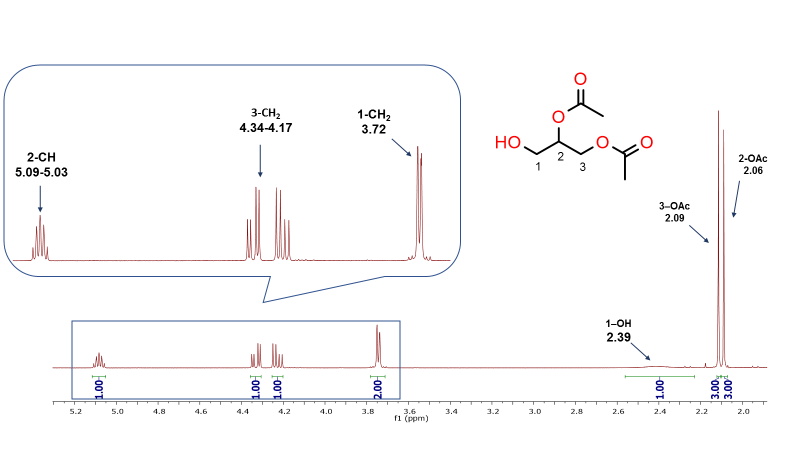


Figure S3. EI MASS SPECTRUM OF 1,2-DIACETIN (2).


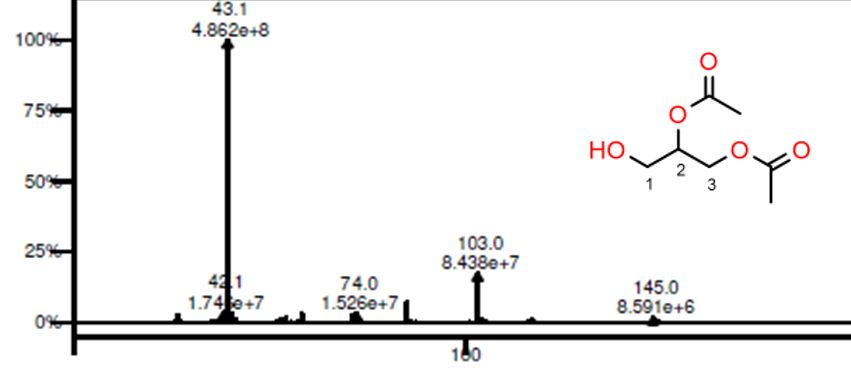


Figure S4. EI MASS SPECTRUM AND PROPOSED FRAGMENTATION PATTERNS OF GLYCERALDEHYDE DIACETATE (3).


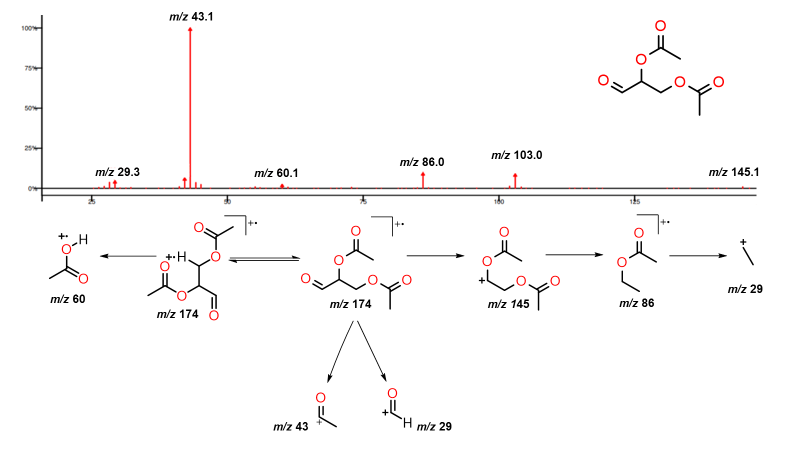


Figure S5. ^1^H NMR SPECTRUM OF 3-((2,3-DIACETOXYPROPANOYL)OXY)PROPANE-1,2-DIYL DIACETATE (5).

**
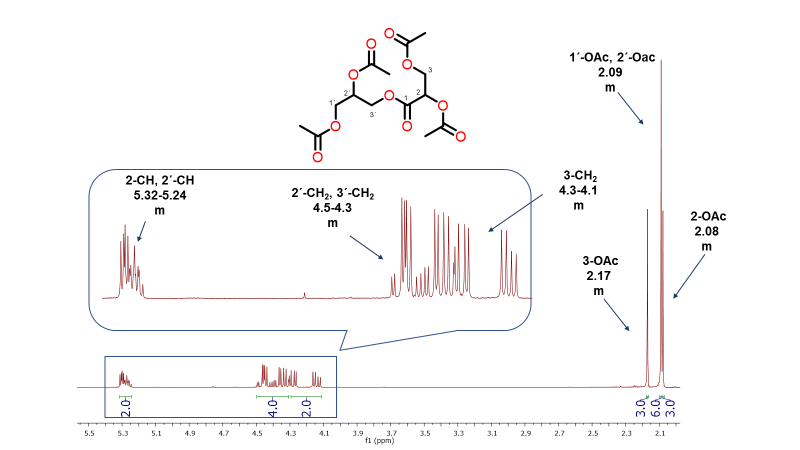
**

Figure S6. ^13^C NMR SPECTRUM OF 3-((2,3-DIACETOXYPROPANOYL)OXY)PROPANE-1,2-DIYL DIACETATE (5).


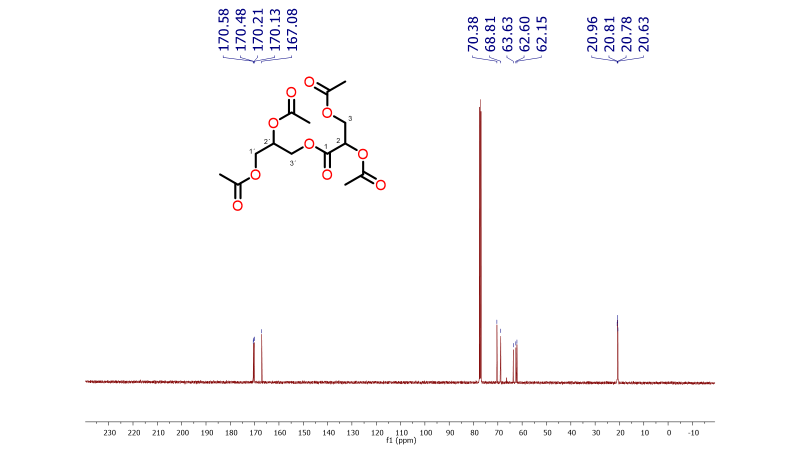


Figure S7. EI MASS SPECTRUM OF 3-((2,3-DIACETOXYPROPANOYL)OXY)PROPANE-1,2-DIYL DIACETATE (5)


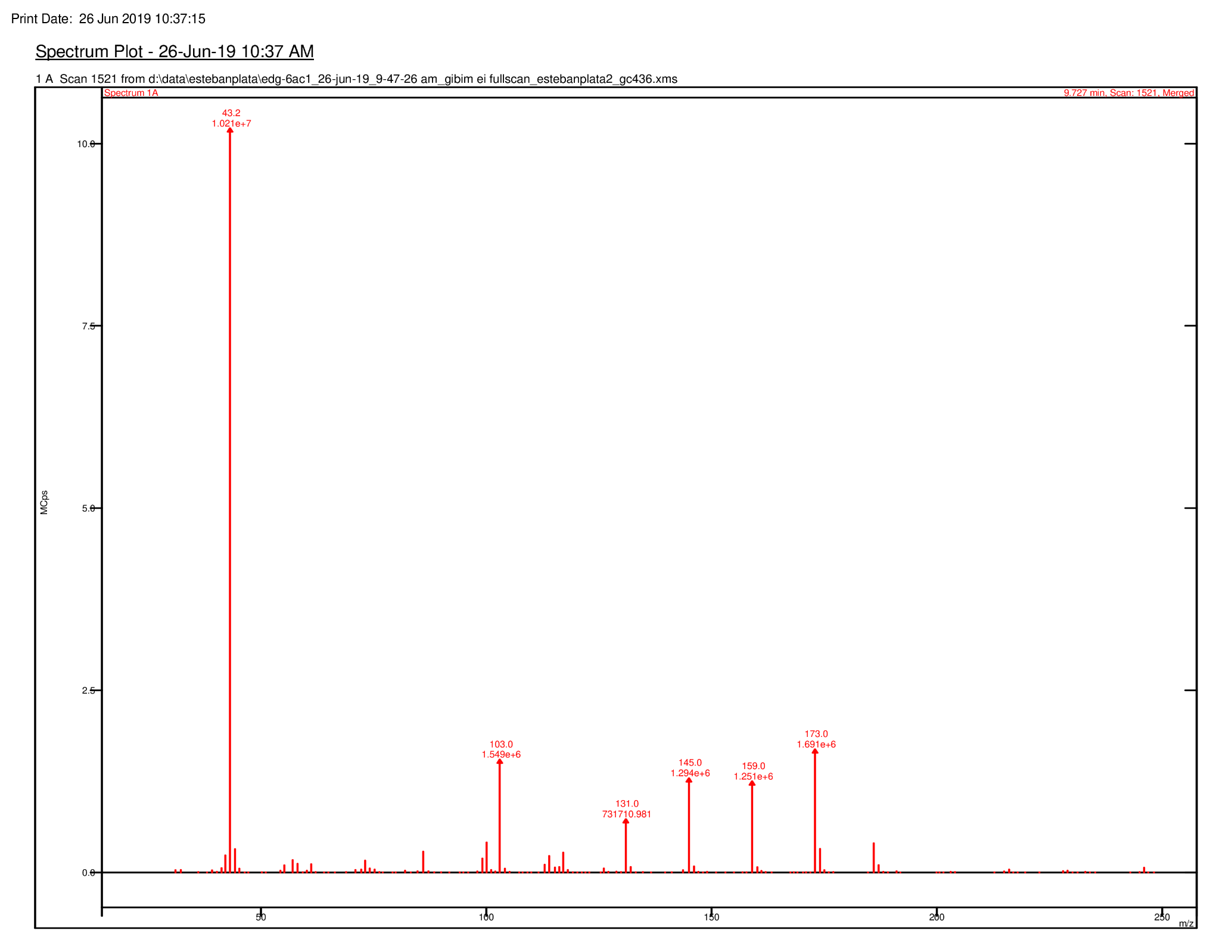


Figure S8. HRMS OF 3-((2,3-DIACETOXYPROPANOYL)OXY)PROPANE-1,2-DIYL DIACETATE (5) C_14_H_21_O_10_


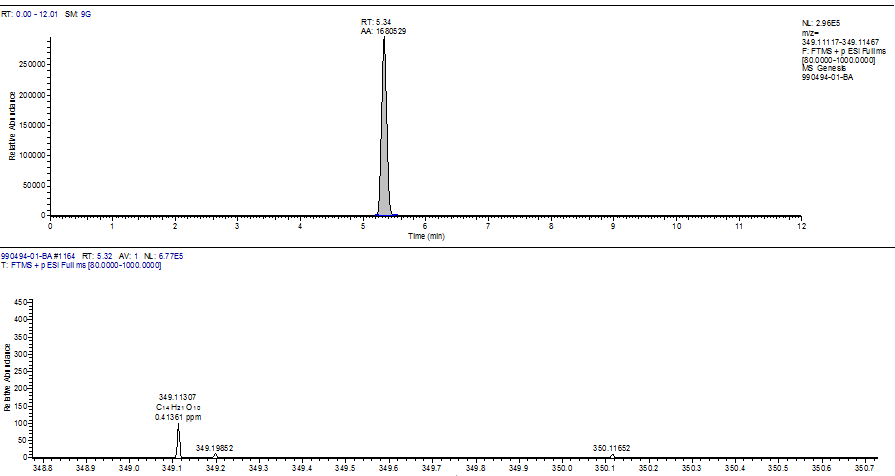


Figure S9. HRMS OF 3-((2,3-DIACETOXYPROPANOYL)OXY)PROPANE-1,2-DIYL DIACETATE (5) C_14_H_24_O_10_N

Figure S10. GAS CHROMATOGRAM AND MASS SPECTRA OF 1,2-DIACETIN OXIDATION WITH PCC ON A ZB-5MS 15 m x 0.25 mm x 1.0 µm COLUMN.


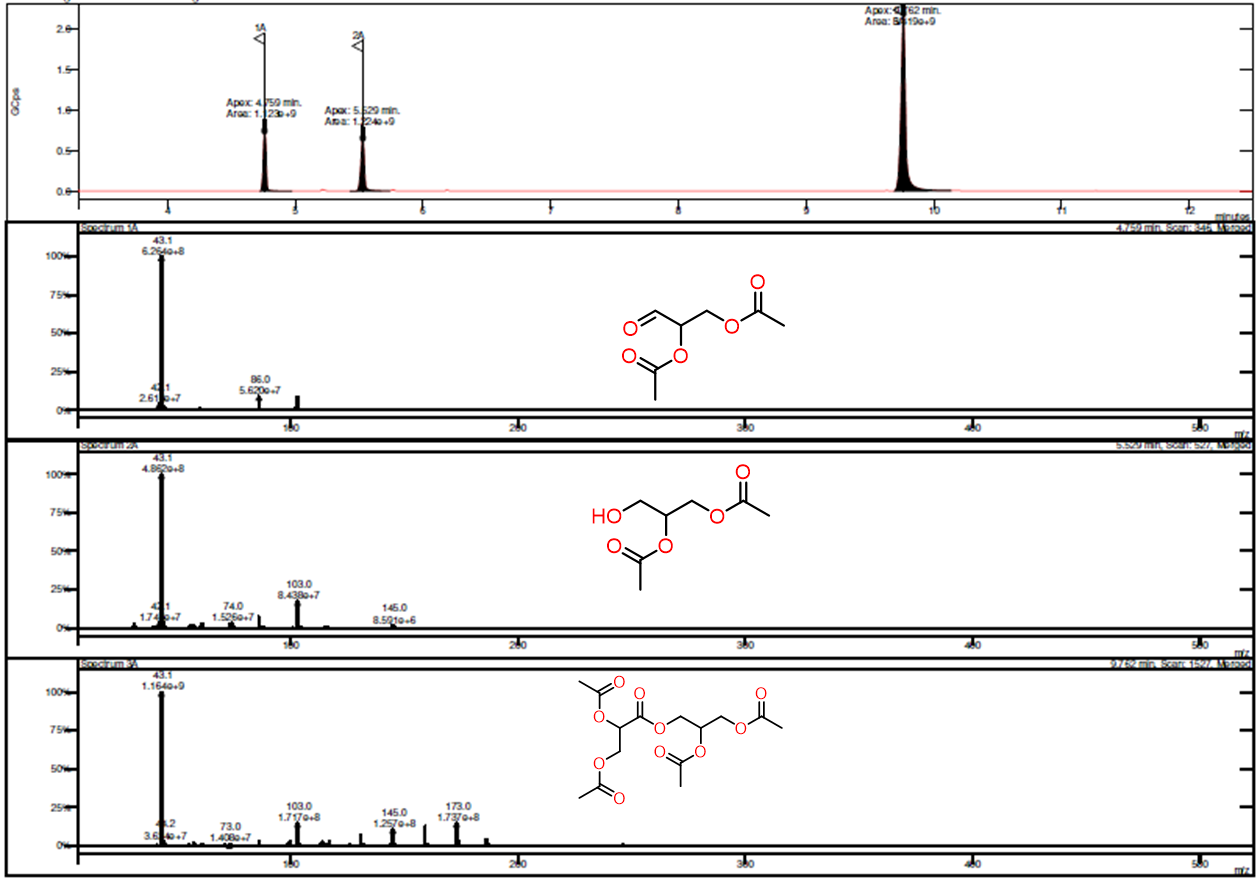


Figure S11. GAS CHROMATOGRAM AND MASS SPECTRA OF 1,2-DIACETIN OXIDATION WITH PCC ON A ZB-5MS 30 m x 0.25 mm x 1.0 µm COLUMN.


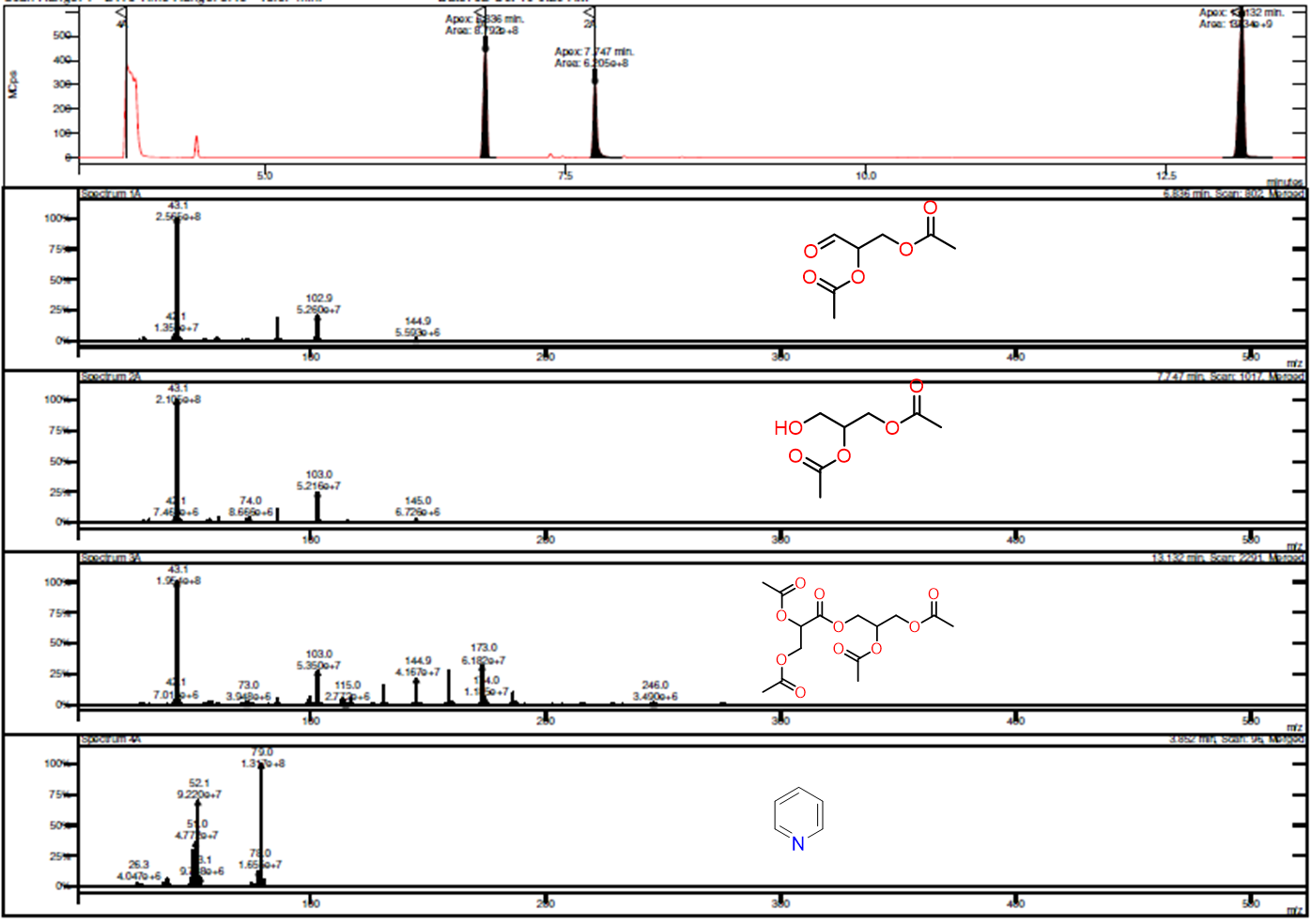


Figure S12. GAS CHROMATOGRAM OF 1,2-DIACETIN OXIDATION WITH 2 EQUIVALENTS OF PCC(TABLE 2 ENTRY 1). ZB-5MS 15 m x 0.25 mm x 1.0 µm COLUMN.


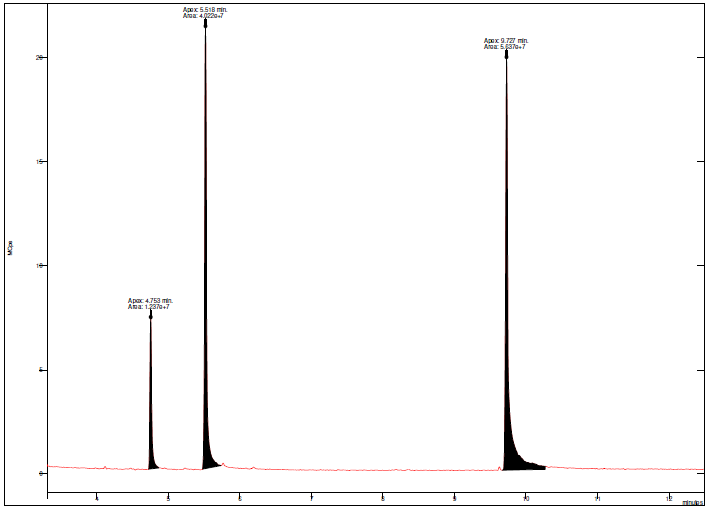


Figure S13. GAS CHROMATOGRAM OF 1,2-DIACETIN OXIDATION WITH 2 EQUIVALENTS OF PCC AND SODIUM ACETATE (TABLE 2 ENTRY 2). ZB-5MS 15 m x 0.25 mm x 1.0 µm COLUMN.


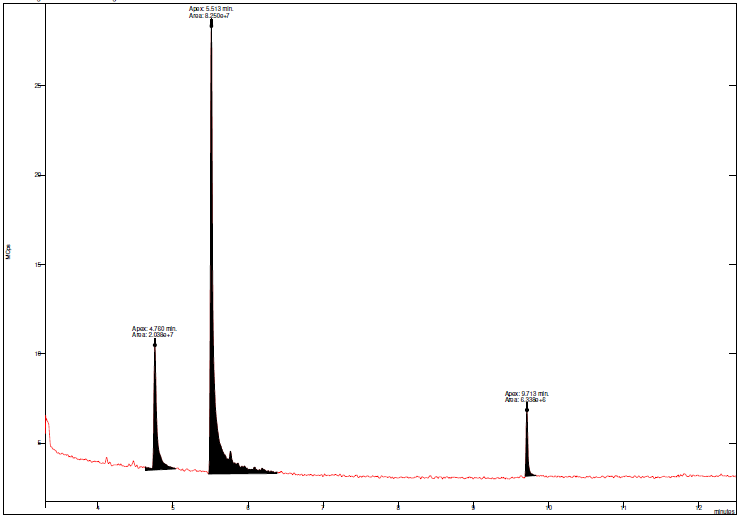


Figure S14. GAS CHROMATOGRAM OF 1,2-DIACETIN OXIDATION WITH 2 EQUIVALENTS OF PCC IN 20 mL/g PCC of CH_2_Cl_2_(TABLE 2 ENTRY 3). ZB-5MS 30 m x 0.25 mm x 1.0 µm COLUMN.


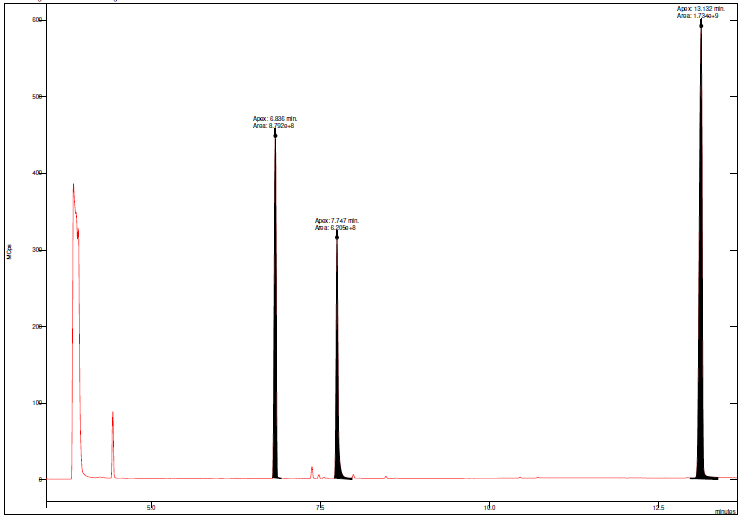


Figure S15. GAS CHROMATOGRAM OF 1,2-DIACETIN OXIDATION WITH 2 EQUIVALENTS OF PCC AND 1 EQUIVALENT OF SODIUM ACETATE IN 20 mL/g PCC of CH_2_Cl_2_(TABLE 2 ENTRY 4). ZB-5MS 30 m x 0.25 mm x 1.0 µm COLUMN.


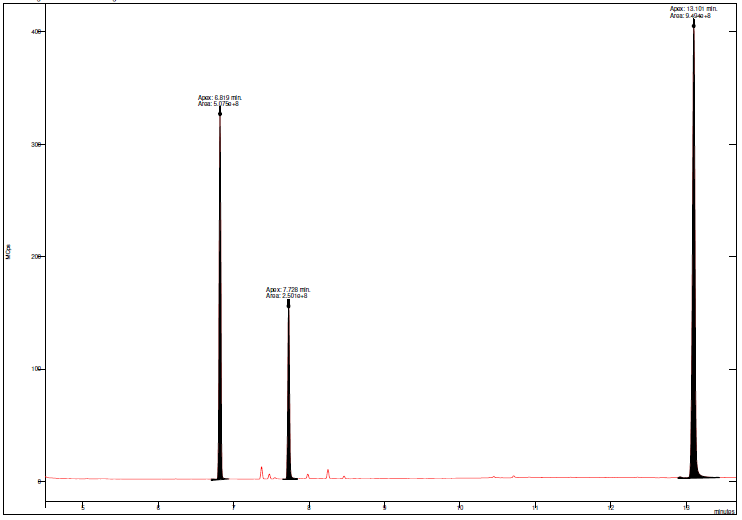


Figure S16. GAS CHROMATOGRAM OF 1,2-DIACETIN OXIDATION WITH 2 EQUIVALENTS OF PCC AND 1 EQUIVALENT OF SODIUM ACETATE AND 2 G OF SILICA GEL IN 20 mL/g PCC of CH_2_Cl_2_(TABLE 2 ENTRY 5) ZB-5MS 15 m x0.25 mm x 1.0 µm COLUMN.


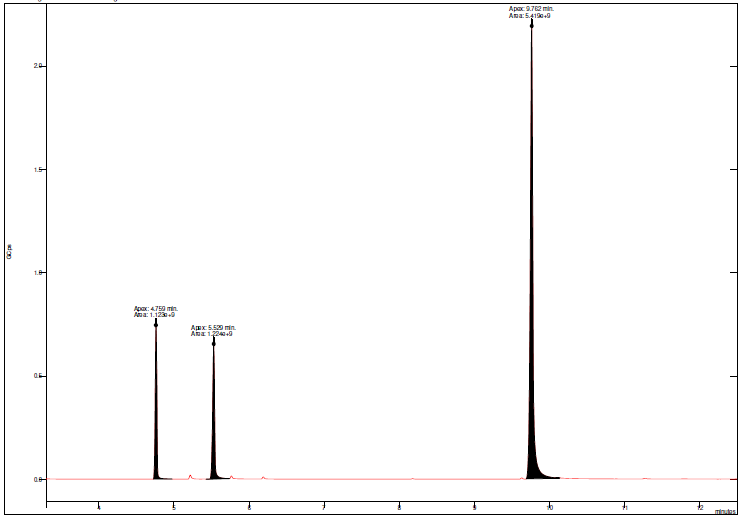


Figure S17. ANTIBACTERIAL ACTIVITY OF 3-((2,3-DIACETOXYPROPANOYL)OXY)PROPANE-1,2-DIYL DIACETATE (5).

| **Strain** | **Growth inhibition (100 ppm)** |
| --- | --- |
| SARM | 17.4 ± 0.5% |
| *S. aureus* ATCC 29213 | 7.7 ± 3.5% |
| *E. coli* ATCC 25922 | -5.3 ± 6.6% |
| *E. coli* O157:H7 | -18.0 ± 2.9% |
| *S*. typhimurium ATCC 14028 | -6.9 ± 8.9% |
| *S.* enteritidis ATCC 13076 | -7.5 ± 2.9% |
| *P. aeruginosa* ATCC 27853 | 23.0 ± 6.23% |


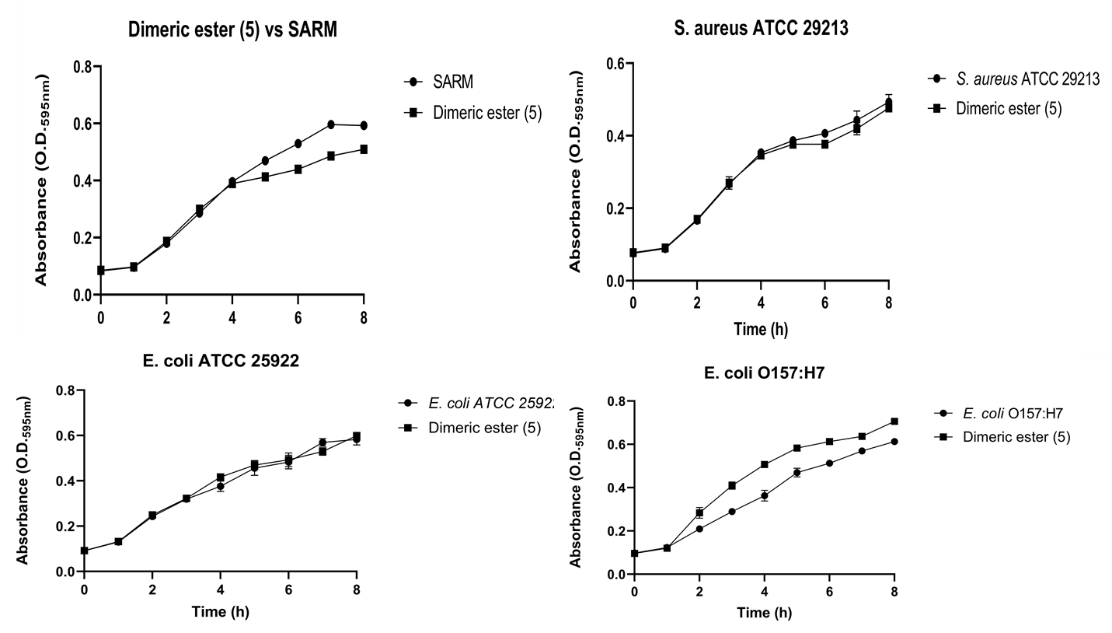

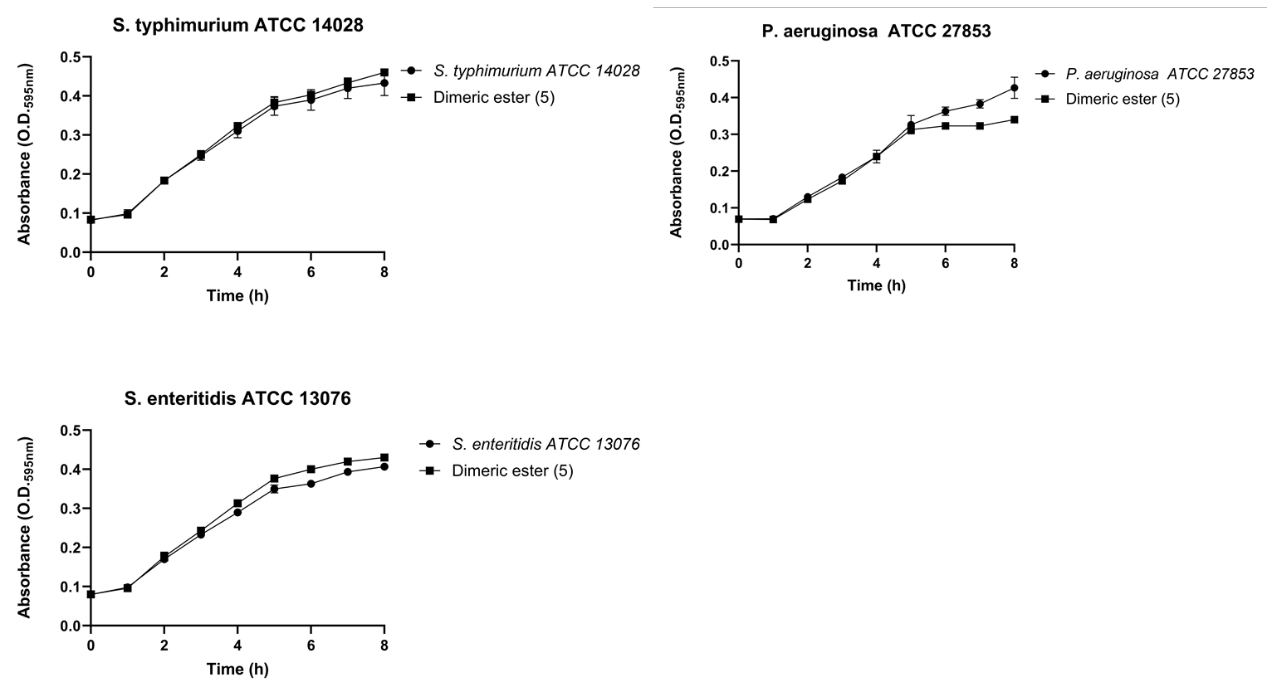


Figure S18. ANTIFUNGAL ACTIVITY OF 3-((2,3-DIACETOXYPROPANOYL)OXY)PROPANE-1,2-DIYL DIACETATE (5).

| **Strain** | **Growth inhibition (100 ppm)** |
| --- | --- |
| *C. albicans* ATCC 10231 | 48.4 ± 1.2% |
| *C. parapsilosis* ATCC 22019 | 67.5 ± 4.3% |


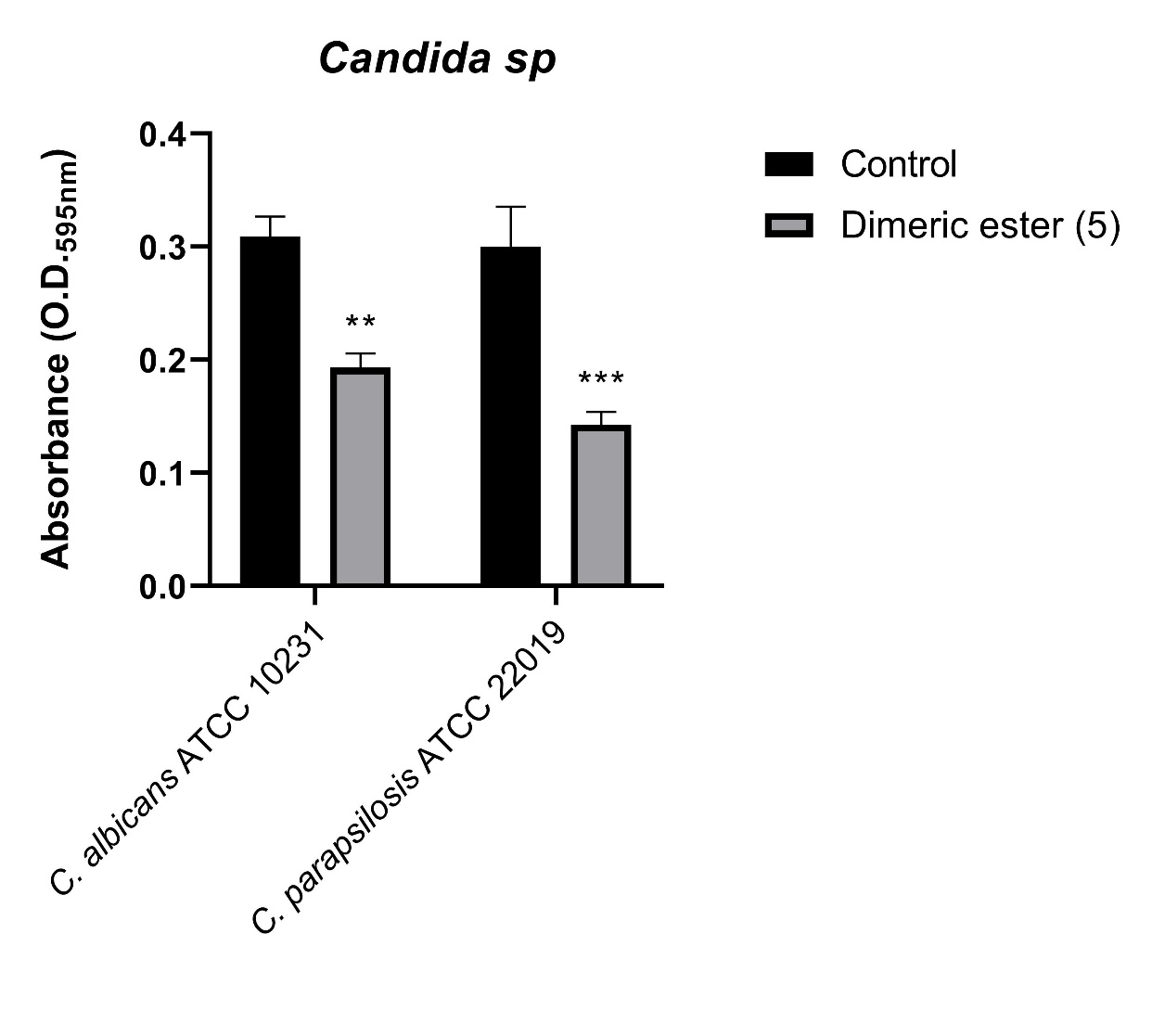


Figure S19. HEMOLYTIC ACTIVITY OF 3-((2,3-DIACETOXYPROPANOYL)OXY)PROPANE-1,2-DIYL DIACETATE (5).


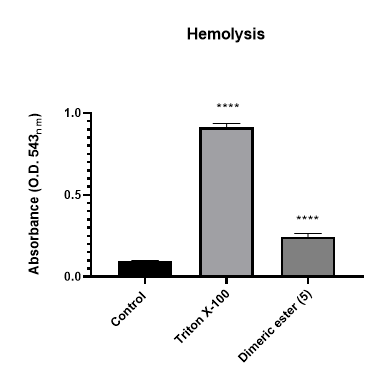

Supplement: Supplementary file 1 [file ijms-21-06501-s001.zip › ijms-904744-supplementary.docx]
